# UPLC-MS/MS Profile of Alkaloids with Cytotoxic Properties of Selected Medicinal Plants of the *Berberidaceae* and *Papaveraceae* Families

**DOI:** 10.1155/2017/9369872

**Published:** 2017-08-30

**Authors:** Anna Och, Katarzyna Szewczyk, Łukasz Pecio, Anna Stochmal, Daniel Załuski, Anna Bogucka-Kocka

**Affiliations:** ^1^Chair and Department of Pharmaceutical Botany, Medical University of Lublin, Chodźki 1, 20-093 Lublin, Poland; ^2^Institute of Soil Science and Plant Cultivation, 8 Czartoryskich, 24-100 Puławy, Poland; ^3^Department of Pharmacognosy, Ludwik Rydygier Collegium Medicum, Nicolaus Copernicus University, 9 Marie Curie-Skłodowska Street, 85-094 Bydgoszcz, Poland; ^4^Chair and Department of Biology and Genetics, Medical University of Lublin, Chodźki 4a, 20-093 Lublin, Poland

## Abstract

Cancer is one of the most occurring diseases in developed and developing countries. Plant-based compounds are still researched for their anticancer activity and for their quantity in plants. Therefore, the modern chromatographic methods are applied to quantify them in plants, for example, UPLC-MS/MS (ultraperformance liquid chromatography tandem mass spectrometry). Therefore, the aim of the present study was to evaluate the content of sanguinarine, berberine, protopine, and chelidonine in *Dicentra spectabilis* (L.) Lem., *Fumaria officinalis* L., *Glaucium flavum* Crantz, *Corydalis cava* L., *Berberis thunbergii* DC., *Meconopsis cambrica* (L.) Vig., *Mahonia aquifolium* (Pursh) Nutt., *Macleaya cordata* Willd., and *Chelidonium majus* L. For the first time, N,N-dimethyl-hernovine was identified in *M. cambrica*, *B. thunbergii*, *M. aquifolium*, *C. cava*, *G. flavum*, and *C. majus*; methyl-hernovine was identified in *G. flavum*; columbamine was identified in *B. thunbergii*; and methyl-corypalmine, chelidonine, and sanguinarine were identified in *F. officinalis* L. The richest source of protopine among all the examined species was *M. cordata* (5463.64 ± 26.3 *μ*g/g). The highest amounts of chelidonine and sanguinarine were found in *C. majus* (51,040.0 ± 1.8 *μ*g/g and 7925.8 ± 3.3 *μ*g/g, resp.), while *B. thunbergi* contained the highest amount of berberine (6358.4 ± 4.2 *μ*g/g).

## 1. Introduction

Different ethnic communities of the world have used plant-based drugs to manage various ailments for centuries. Natural sources of drugs, such as paclitaxel (*Taxus brevifolia*) and *Vinca* alkaloids, are examples for the value of traditionally used plants for modern drug development. Plant-based molecules are used very often as drug precursors being converted into drugs by chemical modification, for example, 10-deacetybaccatin. It is established that about 120 plant-derived compounds are used in western medicine, and about 80% of the world population use medicinal plants in primary health care. Despite the fact that a lot of progress has been made towards the discovery of effective anticancer drugs, many western communities still use the plant-based drugs, including plants from traditional Chinese medicine. The plant-based drugs are used in developed and developing countries separately or together with synthetic drugs [1].

Alkaloids are naturally occurring chemical compounds with the strongest pharmacological activity among substances synthesized by plants. They are responsible for toxic properties of many plant species. High biological activity made these compounds the subject of study for their use in pharmacy, particularly as anticancer drugs. Sanguinarine, berberine, and protopine are quaternary isoquinoline alkaloids, while chelidonine is a tertiary alkaloid. These compounds are extensively investigated for their antitumor activity. According to literature reports, sanguinarine induces apoptosis of many tumor cell lines [2, 3]. Berberine also possesses antitumor activity against several cell lines [4, 5]. Chelidonine is the main alkaloid of great celandine (*Chelidonium majus* L.). An increased interest in chelidonine was observed at the beginning of the 21st century, when scientists began to focus on its promising anticancer activity, such as against uveal melanoma cells [6], liver cancer [7], leukemia cells [8], or melanoma cells [6]. Protopine is an alkaloid with high pharmacological activity. It inhibits blood platelet aggregation and acts antihistaminically and antibacterially [9]. Regarding its antitumor activity, the fact of great importance is that the compound significantly increases the mRNA levels of CYP1A1 in human liver cells and hepatoma HepG2 cells [10]. In prostate cancer cells, the compound inhibits mitosis and induces apoptosis [11]. These alkaloids are present mainly in plant species of *Papaveraceae*, *Rannunculaceae*, and *Berberidaceae* families.

There are different ways of the anticancer alkaloid action, including an induction and activation of the apoptotic signaling proteins and promotion of apoptosis via an induction of DNA damage, caspase activators, and cell growth inhibitors. Apart from these modes of action, there are the other ones based on the formation of G-quadruplexes. This mode of action can be considered as a novel approach for cancer treatment [12, 13].

*C. majus*, *M. cordata*, *D. spectabilis*, *F. officinalis*, *G. flavum*, *C. cava*, *B. thunbergii*, *M. cambrica*, and *M. aquifolium* are the sources of alkaloids. The best known among the examined species are *C. majus* and *M. cordata*, characterized by the highest content of chelidonine. Until now, only these two species have been analyzed using a UPLC method [14]. *B. thunbergii* is the species which is currently arousing a great interest in research, particularly in China. It is the plant of the traditional Chinese and Tibetan medicine where it is highly effective in treatment of many inflammatory diseases in this region. Other species are also used as folk medicine in North America and Europe and as Ayurvedic medicine for their very rich chemical composition [15–17]. Until now, the discussed plant species have been analyzed by methods such as nonaqueous capillary electrophoresis-electrospray ion trap mass spectrometry [18], chromatography on a silica-gel column, TLC [19], and HPLC [18]. For the species *F. officinalis* and *G. flavum*, methods of individual substance extraction were mostly developed [14, 20]. *M. aquifolium* was studied mainly in terms of its antimicrobial activity due to the presence of (4-*O-* methyl-*α*-D-glucurono)-D-xylan possessing potent immunomodulation activity [21].

In this paper, we report on the separation and determination of four isoquinoline alkaloids in nine plant species in order to confirm published data, obtained using other analytical methods. Seven of the investigated species have never been analyzed using UPLC. The study was also conducted in order to identify new alkaloids in the investigated plant species. The UPLC method was chosen as it is precise, accurate, reproducible, and allows the identification of compounds based on their ESI-MS/MS spectra.

## 2. Experimental

### 2.1. Materials and Chemicals

*Berberis thunbergii*, *Chelidonium majus*, *Corydalis cava*, *Dicentra spectabilis*, *Fumaria officinalis*, *Glaucium flavum*, *Macleaya cordata*, *Mahonia aquifolium*, and *Meconopsis cambrica* were collected in June 2012 in the Botanical Gardens of Maria-Curie Sklodowska University in Lublin, Poland. These specimens were authenticated by Professor Anna Bogucka-Kocka (voucher specimens: DS-0612, FO-0612, GF-0612, CC-0612, BT-.0612, MeCa-0612, MA-0612, CM-0612, and MaCo-0612). The reference compounds protopine (P), berberine (B), chelidonine (CH), and sanguinarine (S) were of analytical grade from Sigma-Aldrich Company (St. Louis, USA). The purity of each compound was more than 98%, according to the manufacturer. Acetonitrile, ammonium acetate, methanol gradient HPLC grade, and acetic acid (>98%) for LC-UV-MS separations were purchased from J.T. Baker (Phillipsburg, NJ). Water was purified in-house using a Simplicity-185 with the Milli-Q water purification system (Millipore Co.).

### 2.2. Sample Preparation

Mixed standard stock solutions containing protopine (P) (0.960 mg/ml), berberine (B) (1.105 mg/ml), chelidonine (CH) (1.225 mg/ml), and sanguinarine (S) (1.065 mg/ml) in methanol were prepared and diluted with methanol to six different concentrations within the ranges (P) 4.4–131.5 *μ*g/ml; (B) 6.1–183.8 *μ*g/ml; (CH) 5.5–165.8 *μ*g/ml; and (S) 5.3–159.8 *μ*g/ml for the preparation of calibration curves. The standard solutions were filtered through a 0.22 mm membrane prior to injection. All solutions were stored in a refrigerator at 4°C before analysis.

The extracts were prepared according to the method as described earlier [22]. Five grams of the dried and ground plant materials were macerated with 80 ml of methanol for 3-4 days. Subsequently, methanol was poured off and a new portion was added. This process was repeated nine times. After the last extraction, there was no residue after the evaporation of solvent. The obtained portions of the extracts were combined. The solvent was partially evaporated in the vacuum evaporator UnipanPro Typ 365 to the total volume of 100 ml.

### 2.3. Ultraperformance Liquid Chromatography-Tandem Mass Spectrometry (UPLC-MS/MS)

Chromatographic conditions were based on Lu et al. [12] with some modifications. The UPLC analysis was performed using the Waters ACQUITY UPLC system (Waters Corp., Milford, MA, USA) equipped with a binary pump system, sample manager, column manager, and PDA detector (Waters Corp.). Waters MassLynx software v.4.1 was used for acquisition and data processing. The separation of alkaloids in analyzed extracts was carried out with BEH C18 column (100 mm × 2.1 mm × 1.7 *μ*m), Waters Corp., Milford, MA, USA. Column temperature was maintained at 35°C. The flow rate was adjusted to 0.40 ml/min. Elution was conducted using mobile phase A (ammonium acetate, 20 mM, adjusted to pH 3.0 with acetic acid in Milli-Q water) and mobile phase B (acetonitrile) with gradient program as follows: 0–0.8 min, 3% B; 0.8–1.0 min, 3–12% B; 1.0–9.5 min, 12% B; 9.5–15.0 min, 12–20% B; 15.0–20.0 min, 20–30% B; 20.0–21.0 min, 30% B; 21.0–21.1 min, 30–80% B; 21.1–22.1 min, 80% B; 22.1–22.2, 80–3% B; and 22.2–25.0 min, 3% B. The samples were kept at 15°C in the sample manager. The injection volume of the sample was 1.0 *μ*l (partial loop with needle overfill mode). A strong needle wash solution (95 : 5, methanol-water, *v*/*v*) and a weak needle wash solution (5 : 95, acetonitrile-water, *v*/*v*) were used. Chromatograms were acquired at 240 nm and 270 nm at a 5-point rate, at 4.8 nm resolution. Peaks were assigned on the basis of their retention times, mass to charge ratio (*m/z*), and ESI-MS/MS fragmentation pattern, in comparison to those of the reference compounds and literature data [23]. The MS analyses were carried out on a TQD mass spectrometer (Waters Corp.) equipped with a Z-spray electrospray interface. The parameters for ESI source were capillary voltage 3.0 kV, cone voltage 30 V, desolvation gas N_2_ 800 L/h, cone gas N_2_ 80 L/h, source temp. 120°C, desolvation temp. 350°C, dwell time 0.6 s. Analysis was carried out using a full scan mode (mass range of 250–450 amu was scanned). Compounds were analyzed in a positive ion mode. The content of identified compounds was estimated based on the peak area at 270 nm [+/− very low content (below 5% of the total peak area at 270 nm), + low content (below 15% of the total peak are at 270 nm), ++ moderate content (15–30% of the total peak area at 270 nm), and +++ high content (above 30% of the total peak area at 270 nm)]. The protopine, berberine, chelidonine, and sanguinarine were quantified on the basis of their peak areas and comparison with a calibration curve obtained with the corresponding standards.

The methods were validated in terms of accuracy, precision LOD, and LOQ. Moreover, the linear ranges of calibration curves were determined. The stock solution of the four standards was prepared and diluted to six appropriate concentrations for the establishment of calibration curves. The regression equations were achieved after linear regression of the peak areas versus the corresponding concentrations. Limit of detection (LOD) and limit of quantification (LOQ) for each analyte were determined under the chromatographic conditions at a signal-to-noise ratio (S/N) of 3 and 10, respectively. Intraday and interday variations were chosen to determine the precision of the developed assay. Three different concentration solutions (low, medium, and high) of the standards were prepared. The intraday variation was determined by analyzing seven replicates a day. Interday variation was examined in seven days. Repeatability was confirmed with six solutions prepared from sample 1 and one of them was injected into the apparatus at 0, 3, 6, 12, 16, 18, and 24 h to access the stability of the solution. Variations were expressed by RSDs.

## 3. Results and Discussion

**Figure 1:**
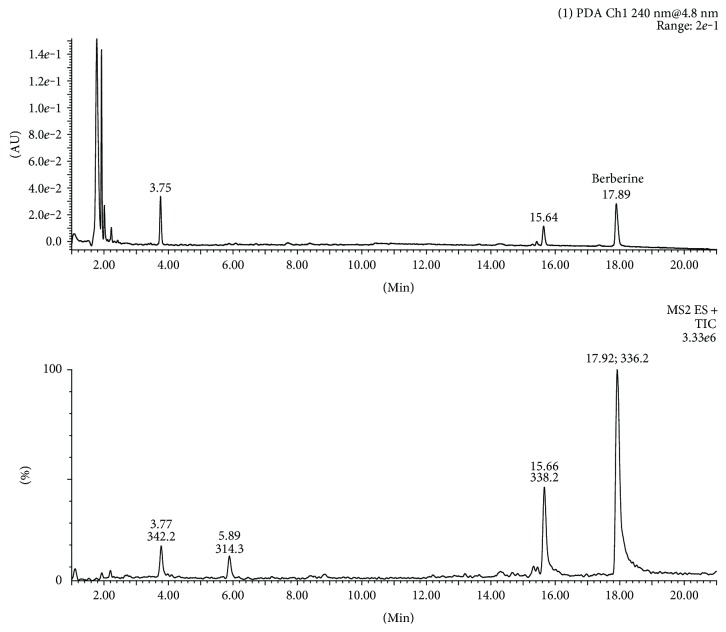
The UPLC-PDA (240 nm) and UPLC-ESI-MS/MS (positive ion mode) chromatograms of *Berberis thunbergii* extract.

In our experiment, the established analytical method was applied for determination of alkaloids in nine species belonging to *Berberidaceae* and *Papaveraceae* families. The analysis of samples revealed the presence of nineteen alkaloids. For the final identification of B, CH, P, and S, the comparisons of retention times with available chemical standards were made. The identification of alkaloids other than B, CH, P, and S was based on the detected maxima and the shape of the spectra. The results are presented in Table 1. Exemplary UPLC chromatograms of *B. thunbergii* extract are shown in Figure 1.

The least differentiated taxa are *M. cambrica* and *D. spectabilis*—containing two of the identified alkaloids. The richest are *C. majus*—eight identified alkaloids and *C. cava*—seven identified alkaloids.

The lowest level of protopine was detected in *M. cambrica* (74.4 ± 0.4 *μ*g/g), while the highest content of it was detected in *M. cordata* (54,636.4 ± 2.6 *μ*g/g). In *M. aquifolium* and *B. thunbergii*, protopine was not detected. Berberine was detected in four of investigated plant species—*C. majus*, *M. cordata*, *B. thunbergii*, and *M. aquifolium*. The lowest level of berberine was detected in *M. cordata* (1401.4 ± 2.4 *μ*g/g), while the highest in *B. thunbergii* (6358.4 ± 4.2 *μ*g/g).

The lowest level of sanguinarine was detected in *C. cava* (69.0 ± 0.1 *μ*g/g), while the highest content of sanguinarine was detected in *C. majus* (7925.8 ± 3.3 *μ*g/g). In *B. thunbergii*, *M. cambrica*, and *M. aquifolium*, sanguinarine was not detected. Chelidonine was detected only in *F. officinalis* (650.0 ± 0.5 *μ*g/g) and *C. majus* (51,040.0 ± 1.8 *μ*g/g). *N,N*-Dimethyl-hernovine was not detected earlier in any of the investigated plants. The compound was observed in *M. cambrica*, *B. thunbergii*, *M. aquifolium*, *C. cava*, *G. flavum*, and *C. majus*. Methyl-hernovine was detected for the first time in *G. flavum* and columbamine in *B. thunbergii*. According to literature data, columbamine occurs in *C. cava* [24] and *M. aquifolium* [25] which was not confirmed in this study. Methyl-corypalmine was detected in *F. officinalis* for the first time. In this study, the presence of fumarophycine [26] and corydamine [27] in this plant material was confirmed. The presence of jatrorrhizine in *M. aquifolium* [25] and corydaline in *C. cava* [28, 29] was also confirmed, but not in *F. officinalis* [27]. Corydine and tetrahydropalmatine were found in this study only in *C. cava* but were not in other investigated plant species. Allocryptopine was detected only in *C. majus* and *M. cordata*. The analysis confirmed the presence of cryptopine in *Chelidonium majus* but not in *F. officinalis* [27] nor in *M. cordata* [30]. The study did not confirm the presence of columbamine in *C. cava* [23] and *M. aquifolium* [25] and cryptopine in *F. officinalis* [27], *D. spectabilis* [31], and *M. cordata* [30].

Validation of the UHPLC method was performed in terms of accuracy and precision LOD and LOQ. The results of the regression indicated that all four reference compounds showed good linearity in a relatively wide concentration range. The correlation coefficients of all calibration curves were *R*^2^> 0.9992. The LODs and LOQs of the four analytes were 10.0–47.8 and 33.2–159.6 *μ*g/g, respectively. Parameters of calibration curves together with LOD and LOQ values are presented in Table 2.

The relative standard deviation (RSD%), as a measure of repeatability, was from 0.58% (berberine) to 1.25% (chelidonine). These values are in good agreement with requirements for a developed method. The intra- and interday precision RSD values were less than 3.0% for all compounds, which showed good reproducibility of the method. The results are summarized in Table 3.

## 4. Conclusions

Despite the extensive research conducted on the phytochemical composition of the investigated species, several alkaloids were detected in this study for the first time. These were cryptopine in *Chelidonium majus*; N,N- dimethyl-hernovine in *Meconopsis cambrica*, *Berberis thunbergii*, *Mahonia aquifolium*, *Corydalis cava*, *Glaucium flavum*, and *Chelidonium majus*; methyl-hernovine in *Glaucium flavum*; columbamine in *Berberis thunbergii*; methyl-corypalmine, chelidonine, and sanguinarine in *Fumaria officinalis*; and sanguinarine in *Corydalis cava*. Environmental conditions are one of the possible causes of variation of alkaloidal composition of the tested plant materials, since the data were obtained from studies of the raw materials originating from Turkey and China. These differences may also result from the analytical methods used, because extracts from *C. cava* and *F. officinalis* were not analyzed by UPLC-MS/MS method earlier.

## Figures and Tables

**Table 1:** The contents (*μ*g/g) of alkaloids in investigated plant species.

Plant species	Compound																		
	Allocryptopine	Berberine^∗^	Chelerythrine	Chelidonine^∗^	Columbamine	Coptisine	Corydaline	Corydamine	Corydine	Cryptopine	*N,N*-Dimethyl-hernovine	Fumarophycine	Glaucine	Jatrorrhizine	Methyl-corypalmine	Methyl-hernovine	Protopine^∗^	Sanguinarine^∗^	Tetrahydropalmatine
*Berberis thunbergii*	−	6358.4 ± 4.2	−	−	++	−	−	−	−	−	+/−	−	−	−	−	−	−	−	−
*Chelidonium majus*	+++	5366.6 ± 4.3	+++	51,040.0 ± 1.8	−	−	−	−	−	++	+++	−	−	−	−	−	11,660.6 ± 13.6	7925.8 ± 3.3	−
*Corydalis cava*	−	−	−	−	−	++	++	−	++	−	++	−	−	−	−	−	1764.0 ± 0.7	69.0 ± 0.1	+/−
*Dicentra spectabilis*	−	−	−	−	−	−	−	−	−	−	−	−	−	−	−	−	11,899.6 ± 8.6	1105.6 ± 1.0	−
*Fumaria officinalis*	−	−	−	650.0 ± 0.5	−	−	−	+/−	−	−	−	+/−	−	−	+/−	−	3726.6 ± 0.6	125.4 ± 0.2	−
*Glaucium flavum*	−	−	−	−	−	−	−	−	−	−	++	−	+++	−	−	+/−	4990.0 ± 1.3	407.4 ± 0.9	−
*Macleaya cordata*	+++	1401.4 ± 2.4	−	−	−	−	−	−	−	−	−	−	−	−	−	−	54,636.4 ± 2.6	1835.0 ± 4.3	−
*Mahonia aquifolium*	−	3344.6 ± 3.7	−	−	−	−	−	−	−	−	++	−	−	++	−	−	−	−	−
*Meconopsis cambrica*	−	−	−	−	−	−	−	−	−	−	+	−	−	−	−	−	74.4 ± 0.4	−	−

^∗^Comparisons with chemical standard have been made; without ∗ indicates tentative assignment based on UV-Vis and MS/MS profile. −: not detected; +/−: tentatively detected, <5% based on the peak area recorded at 270 nm for all identified; +: <15% based on peak area recorded at 270 nm for all identified, ++: 15–30% based on the peak area recorded at 270 nm for all identified; +++: >30% based on the peak area recorded at 270 nm for all identified.

**Table 2:** Analytical parameters of UPLC-MS/MS quantitative methods; data for calibration curves, limit of detection (LOD), and limit of quantification (LOQ) values for four analyzed alkaloids. *y* = *Ax* + *B*, where *y* is the peak area, *x* is concentration of the alkaloids (*μ*g/g), and *R*^2^ is the correlation coefficient of the equation.

Analytes	Regression equations	*R* ^2^	Linear range (*μ*g/g)	LOD (*μ*g/g)	LOQ (*μ*g/g)
Protopine	*y* = −129.20 + 58.62*x*	0.9992	88–2630	14.0	46.6
Berberine	*y* = −607.10 + 133.00*x*	0.9992	110–3316	47.8	159.6
Chelidonine	*y* = −35.14 + 53.91*x*	0.9996	122–3676	12.6	42.0
Sanguinarine	*y* = −344.30 + 167.20*x*	0.9997	106–3196	10.0	33.2

**Table 3:** Precision, repeatability, and stability of four alkaloids in *Ch. majus* L.

Analytes	Precision			Repeatability (RSD%)
	Levels (*μ*g/g)	Intraday RSD (%)	Interday RSD (%)	
Protopine	88	0.63	2.43	0.64
	1680.0	0.40	0.56	
	2620.0	0.62	0.28	

Berberine	140	0.62	0.43	0.58
	1800.0	0.97	1.80	
	3660	0.88	0.79	

Chelidonine	110	0.67	1.40	1.25
	1100.0	0.25	0.63	
	3300.0	0.63	0.99	

Sanguinarine	106	0.62	1.36	0.80
	1960.0	0.96	1.15	
	3180.0	0.36	0.84	

## Abbreviations

P: Protopine
B: Berberine
CH: Chelidonine
S: Sanguinarine
UPLC-MS/MS: Ultraperformance liquid chromatography-tandem mass spectrometry.
